# Express yourself: how PP2A-B55^Pab1^ helps TORC1 talk to TORC2

**DOI:** 10.1007/s00294-017-0721-8

**Published:** 2017-06-22

**Authors:** Ruth Martín, Sandra Lopez-Aviles

**Affiliations:** Centre for Molecular Medicine Norway, Nordic EMBL Partnership, University of Oslo, Gaustadalleen 21, 0349 Oslo, Norway

**Keywords:** TORC1, TORC2, PP2A-B55, Gad8, Nitrogen signaling, Differentiation

## Abstract

The control of cell fate, growth and proliferation in response to nitrogen availability is a tightly controlled process, with the two TOR complexes (TORC1 and TORC2) and their effectors playing a central role. PP2A-B55^Pab1^ has recently been shown to be a key element in this response in fission yeast, where it regulates cell cycle progression and sexual differentiation. Importantly, a recent study from our group has shown that PP2A-B55^Pab1^ acts as a mediator between the activities of the two TOR signaling modules, enabling a crosstalk that is required to engage in the differentiation program. In this review, we recapitulate the studies that have led to our current understanding of the interplay between TOR complexes. Moreover, we discuss several aspects of the response to nitrogen availability that still require further attention, and which will be important in the future to fully realize the implications of phosphatase activity in the context of TOR signaling.

## Introduction

Since the discovery in the early 90s of the target of rapamycin, research in the field of TOR signaling has come a long way. In this time the two TOR complexes, TORC1 and TORC2, have taken centre stage as main players in the regulation of cell growth, differentiation and proliferation. We owe a great part of our current understanding of their function and regulation to the research conducted in the baker’s yeast *Saccharomyces cerevisiae* (reviewed in Loewith and Hall 2011). Studies in the fission yeast *Schizosaccharomyces pombe* have followed closely behind and, ever since their identification in this model organism, they have rendered important advances in the field. The two distant yeast species share the same particularity of containing two different catalytic subunits, which has facilitated the dissection of the individual roles of TORC1 and TORC2.

In fission yeast, rather confusingly, TORC1 contains the catalytic subunit Tor2 (and in some instances Tor1 (Hartmuth and Petersen 2009)), whereas TORC2 contains Tor1 (reviewed in Kanoh and Yanagida 2010). Early work in fission yeast revealed that these two complexes play opposite roles during the differentiation response that occurs upon nitrogen starvation. While inactivation of TORC1 leads to cell shortening, cell cycle arrest in G1 and induction of differentiation genes (similar to the response to nitrogen depletion) (Alvarez and Moreno 2006; Uritani et al. 2006; Weisman et al. 2006; Matsuo et al. 2007), deletion of *tor1*+ or its substrate *gad8*+ leads to an elongated cell phenotype and inability to arrest in G1 and to express differentiation genes upon nitrogen starvation (Weisman and Choder 2001; Weisman et al. 2006). Hence, while TORC1 has to become inactivated during the nitrogen starvation response, the activity of TORC2 and Gad8 has to be preserved to engage into sexual differentiation. Perhaps this is the clearest example of the two TOR complexes exerting opposite roles in a given process. In this review, we revisit the functions of TORC1 and TORC2 and their complex relation, from yeast to mammals. Moreover, we focus on recent discoveries in fission yeast involving the protein phosphatase PP2A-B55^Pab1^ and we look at the different directions that these findings will lead us to.

## Shared and opposite roles of TORC1 and TORC2

In mammalian systems the general idea is that TORC1 and TORC2 work in a coordinated manner to promote growth and proliferation. TORC1 has been implicated in almost every aspect of cell anabolism, from protein translation to lipogenesis and nucleic acid synthesis (reviewed in Shimobayashi and Hall 2014). Its role as an inhibitor of autophagy through multiple targets is also well documented (reviewed in Russell et al. 2014). TORC2 favors cell survival and metabolism through the activation of AKT and SGK1 (Sarbassov et al. 2005; García-Martínez and Alessi 2008). It also regulates the actin cytoskeleton (Cybulski and Hall 2009), and new evidences indicate that, in parallel to TORC1, it promotes lipogenesis by activating SERBP (Hagiwara et al. 2012; Yuan et al. 2012). More extensive studies are still needed but, since TORC2 is activated by direct association to ribosomes (Zinzalla et al. 2011), a role during protein translation would also be expected.

Nevertheless, TORC1 and TORC2 have been shown to have opposite roles during the differentiation of specific cell types in mammalian cells. During mesenchymal stem cell differentiation, loss of TORC1 or TORC2 activity (mediated by the depletion of Raptor or Rictor) leads to different outcomes. Thus, while deletion of Raptor impairs adipogenic differentiation and enhances osteogenic differentiation, deletion of Rictor has the opposite effect (Martin et al. 2015). Similarly, T cells lacking TORC1 activity fail to differentiate into T_H_1 or T_H_17, while their ability to differentiate into T_H_2 is enhanced. In contrast, deletion of Rictor hinders T_H_2 differentiation with no obvious effects on the differentiation of T_H_1 and T_H_17 lineages (Delgoffe et al. 2011). Another example of the opposite effects of the two TOR complexes is found in mouse models of axon regeneration. In this context, mTORC1 activity is required for axon growth, whereas mTORC2 and GSK3β are inhibitory (Miao et al. 2016).

A study in *C. elegans* has also revealed an antagonistic role of TORC1 and TORC2, in this case during epidermal cellular morphogenesis induced by semaphorin. This signal promotes a shift in the TOR adaptor (from Rictor to Raptor) to enhance TORC1 activity in detriment of TORC2. Failure to favor the balance towards TORC1 brings about epidermal defects that can be rescued by specific inhibition of TORC2 or enhancement of TORC1 (Nukazuka et al. 2011).

Last but not least, in budding yeast TORC2 has been shown to promote autophagy by regulating mitochondrial function. This ultimately leads to the inhibition of Calcineurin, an inhibitor of autophagy (Vlahakis et al. 2014, 2017). This is in clear contrast to the negative effect of TORC1 in this process (reviewed in Kamada and Ohsumi 2010). All in all, these different studies highlight the complexity of TOR signaling and expose how these complexes coordinate their activities to bring about specific responses depending on the cellular context.

## Crosstalk between TORC1 and TORC2

Notably, in mammalian systems, where the regulation of TOR signaling has been extensively studied, multiple mechanisms of feedback and crosstalk between the two TOR complexes have been revealed. Both TORC1 and TORC2 lie downstream of PI3K and the insulin/IGF receptor (among other growth factor receptors). In the case of TORC1, stimulation of PI3K leads to the phosphorylation of AKT on its activation loop (Thr308) by PDK1 (reviewed in Pearce et al. 2010). Active AKT can subsequently phosphorylate TSC2 in multiple sites, blocking its GAP activity towards RHEB, the small GTPase responsible for TORC1 activation (Manning et al. 2002; Inoki et al. 2002; Potter et al. 2002). AKT also phosphorylates PRAS40, a TORC1 inhibitor, to promote its dissociation from TORC1 during insulin stimulation (Vander Haar et al. 2007). In addition, TORC1 requires amino acid sensing for its Rag-dependent recruitment to the lysosomal surface, where it can be activated by RHEB (reviewed in Betz and Hall 2013). Interestingly, in budding yeast mutants hypersensitive to Hygromycin B show defective late endosome-dependent vacuolar trafficking and TORC1 activation (Ejzykowicz et al. 2016). TORC2 also becomes activated in response to growth factors through its PI3K-dependent association to active ribosomes (Zinzalla et al. 2011). TORC2’s best-characterized substrate is AKT itself. On the one hand, TORC2 phosphorylates AKT co-translationally on Thr450 (on the turn motif) to promote its stability (Oh et al. 2010). On the other hand, it also phosphorylates AKT on Ser473 (Sarbassov et al. 2005), which is required for maximal activation of the protein (Alessi et al. 1996). Phosphorylation of AKT Ser473 by TORC2 is not required for TSC2 or PRAS40 phosphorylation (phosphorylation of Thr308 by PDK1 is sufficient for these events). Instead, it allows the phosphorylation of additional substrates (e.g. FOXO proteins) (Guertin et al. 2006). Nevertheless, TORC2 co-translationally phosphorylates IMP1 (IGF2 mRNA-binding protein1), leading to the increased production of IGF2 and enhanced IGFR stimulation (Dai et al. 2013). Thus, TORC2 activation reinforces its own and TORC1’s signaling. In contrast, TORC1 activation has the opposite effect. It has been shown that p70S6K downstream of TORC1 can phosphorylate and inhibit the insulin receptor substrate IRS1 (Harrington et al. 2004) and that sustained activation of TORC1 leads to depletion of IRS1 and IRS2 and insulin resistance (Shah et al. 2004). Moreover, two independent phosphoproteomic analyses of TORC1 substrates identified Grb10 (Growth receptor bound protein 10) as a direct TORC1 substrate (Hsu et al. 2011; Yu et al. 2011). Phosphorylation of Grb10 also leads to IGF signaling inhibition. In addition, a crosstalk between TORC1 and TORC2 has also been described: p70S6K, downstream of TORC1, can phosphorylate the TORC2 component Sin1, promoting its dissociation from the complex and inhibiting its activity (Liu et al. 2013, 2014). Nevertheless, the authors of the study also indicated that AKT could phosphorylate those same sites. A recent report indicated that AKT is, indeed, the major kinase to phosphorylate Sin1. However, in this case the result of the phosphorylation was the activation of TORC2, thus creating a positive feedback loop (Humphrey et al. 2013; Yang et al. 2015).

In fission yeast, another example of crosstalk between TORC1 and TORC2 is represented by the transcriptional induction of Isp7 by TORC2, which acts as an activator of TORC1 (Laor et al. 2014). Moreover, work from our laboratory recently described a mechanism by which active TORC1 limits the activity of TORC2 during growth on nitrogen-rich conditions (Martín et al. 2017). These findings involving the protein phosphatase PP2A-B55^Pab1^ are discussed in more detail in the next sections.

## Protein phosphatases in TOR signaling

An important body of work carried out over the years highlighted the importance of phosphatase activity in the mediation of TOR signaling in budding yeast (reviewed in Düvel and Broach 2004). Nevertheless, in fission yeast this aspect of phosphatase function had not been explored in detail. This prompted us to investigate the implications of different protein phosphatases in the context of nitrogen sensing and sexual differentiation.

Protein phosphatases are relatively scarce compared to the number of protein kinases and phosphorylated substrates in the proteome of any organism. To compensate for this discrepancy, protein phosphatases belonging to the PPP family utilize a combinatorial strategy that allows for the recognition of multiple substrates. Thus, the same catalytic subunit can interact with a variety of regulatory subunits to form an active holocomplex. These regulatory subunits bring selectivity by dictating protein localization and substrate binding (reviewed in Janssens et al. 2008). The PPP family of protein phosphatases includes PP1, PP2A, PP2B (Calcineurin), PP4, PP5, PP6 and PP7. In recent years, PP2A has become a central player in the field of cell cycle regulation, particularly with regard to nutritional signaling (Manchado et al. 2010; Gharbi-Ayachi et al. 2010; Mochida et al. 2010; Bontron et al. 2013; Grallert et al. 2015; Chica et al. 2016). PP2A is a heterotrimeric enzyme composed of a scaffolding subunit (A), a catalytic subunit (C) and a regulatory subunit (B). The A and C subunits form the core enzyme, which interacts with a variable B subunit. In human cells, there are two different isoforms of the A and C subunits (termed α and β). The regulatory subunit comprises four different families: B (also known as B55 or PR55), B’ (B56 or PR61), B’’ (PR48/PR72/PR130) and B’’’ (PR93/PR110). In addition, each family contains between two and five different isoforms (reviewed in Shi 2009). While PP2A is highly conserved through evolution, the reduced number of B type subunits both in budding and fission yeast provides a less complex scenario. The budding yeast genome encodes for a single A subunit (Tpd3) and two redundant isoforms of the C subunit (Pph21/Pph22) that interact with either Cdc55 (B/B55) or Rts1 (B’/B56). Similarly, fission yeast PP2A complexes contain a single A subunit (Paa1), one of the two exchangeable C subunits (Ppa2 or Ppa1) and a B subunit belonging to the B/B55 family (Pab1) or to the B’/B56 family (Par1/Par2). In addition to complex assembly, post-translational modifications of the C-subunit and the interaction with a peptidyl-prolyl isomerase (PTPA) are required to achieve the active conformation of the enzyme (Guo et al. 2013). In recent years, the identification of specific motifs that are recognized by the different regulatory subunits (Hertz et al. 2016; Cundell et al. 2016; Godfrey et al. 2017) are shedding light onto the specific roles of the different subcomplexes.

### Tap42 complexes: alternative PP2A structures mediating TORC1 signaling in budding yeast

The first evidence indicating that phosphatase activity could mediate some of the functions of TORC1 arose from studies using conditional alleles of the PP2A-related phosphatase, Sit4, in budding yeast. A temperature sensitive mutant of *sit4* (*sit4*-*102*) displayed phenotypes reminiscent of rapamycin treatment (Fernandez-Sarabia et al. 1992). Sit4, which forms complexes with regulatory subunits of the SAP family (Sap155, Sap190 and Sap185), was found to interact with Tap42, a high copy suppressor of the *sit4*-*102* mutant. Besides Sit4, Tap42 could also associate with the catalytic subunits of PP2A Pph21 and Pph22. This association was found to be independent of other regulatory subunits and, more interestingly, sensitive to the nutritional status or to treatment with rapamycin (being lost upon starvation or TORC1 inhibition) (Di Como and Arndt 1996). Further studies showed that Tap42 is a direct substrate of TORC1 that binds Sit4 and Pph21 and Pph22 when phosphorylated (Jiang and Broach 1999). Nitrogen starvation leads to Tap42 rapid dephosphorylation by PP2A-B55^Cdc55^ and the dissociation of these complexes. Moreover, Tip41 (a Tap42 interacting protein (Jacinto et al. 2001) is also involved in the disruption of Tap42-Sit4 interaction. Tip41 is itself subject to phosphorylation by TORC1, and it can only exert its inhibitory role when dephosphorylated. Tap42 mediates some, but not all, TORC1 functions in budding yeast. In its phosphorylated form it represses the expression of stress genes by blocking the nuclear translocation of Msn2/Msn4. Phosphorylated Tap42 also prevents the Sit4 dependent dephosphorylation and nuclear translocation of Gln3, which is required for the expression of genes in the nitrogen discrimination pathway (NDP) (Beck and Hall 1999). However, later studies showed that the rapamycin-induced expression of NDP genes required the presence of Tap42 (Düvel et al. 2003). These results have led to the hypothesis that, in transcription, the phosphorylated form of Tap42 inhibits Sit4 and Pph21/Pph22, whereas in the hypophosphorylated state it has activation roles.

Tap42 has been poorly studied in fission yeast. The Sit4-related phosphatase, Ppe1, has received more attention, although not as much as its budding yeast counterpart. Initial studies found that a deletion of *ppe1* could be rescued by temperature-sensitive mutations of *ssp1 or ssp2* (Matsusaka et al. 1995), which encode a CAMKK and AMPKα, respectively. Most of the work on Ppe1 has focused on its roles in regulating actin polarity and glucose utilization, but little is known about its relation to TOR signaling. Nevertheless, it was recently shown that Ssp2 integrates nutritional cues to ultimately control TORC1 activity (Davie et al. 2015). Whether Ppe1 is involved in this regulation is not known yet, but it would be an interesting candidate for future studies. Finally, recent work by the Weisman laboratory showed that TORC1 prevents the Ppe1-dependent dephosphorylation of the GATA factor Gaf1, which has implications in the expression of nitrogen stress-induced genes (Laor et al. 2015). These results indicate that in fission yeast as in budding yeast regulation of Ppe1 by TORC1 plays important roles in the response to nutritional cues.

### Ppk18/Igo1/PP2A-B55^Pab1^ pathway: the TORC1-TORC2 connection

**Fig. 1:**
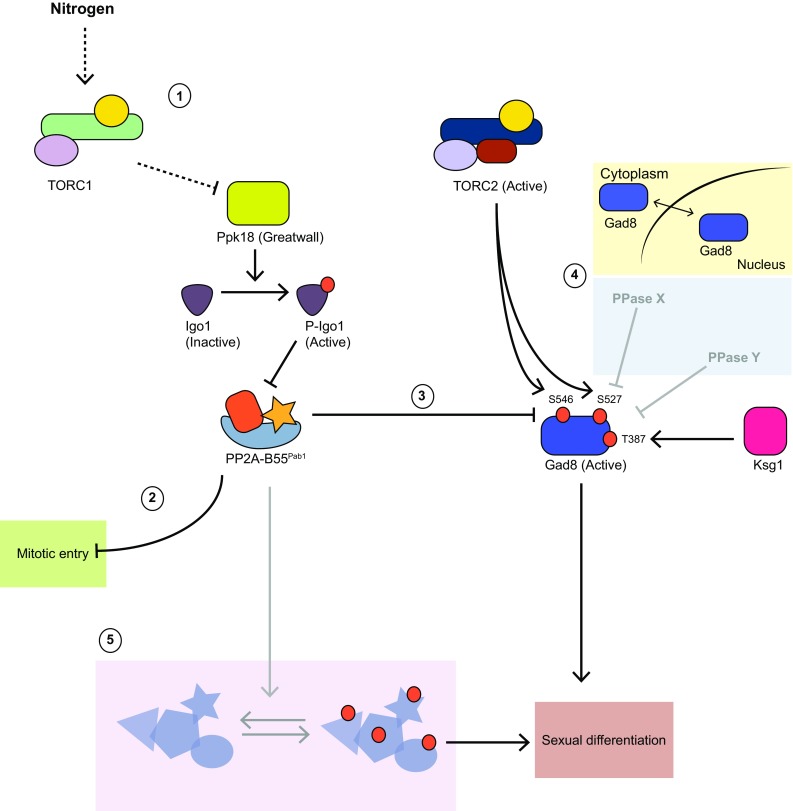
Current understanding of PP2A-B55^Pab1^ regulation and functions and future directions. *Arrows* depicted in *black* represent established events, whereas *arrows* in *grey* indicate potential regulations that would require further investigation (see main text for details). (*1*) PP2A-B55^Pab1^ lies downstream of TORC1 in the response to nitrogen availability, preventing the differentiation response. During growth on nitrogen-rich medium, TORC1 activity leads to the inhibition of Ppk18. In this condition, the Ppk18 substrate Igo1 is not phosphorylated and cannot act as an inhibitor of PP2A-B55^Pab1^. Active PP2A-B55^Pab1^ regulates several events downstream: (*2*) it prevents the G2/M transition, extending the length of the G2 phase and delaying mitotic entry; (*3*) it reverts the phosphorylation of Gad8 by TORC2 on Ser546, preventing sexual differentiation. In response to nitrogen starvation, low TORC1 activity prompts the reactivation of Ppk18 and inhibition of PP2A-B55^Pab1^. In this situation PP2A-B55^Pab1^ can neither exert its repressive role on mitotic entry, nor prevent Gad8 Ser546 hyperphosphorylation. Altogether this leads to cell shortening, arrest in G1 phase and activation of the sexual differentiation program. (*4*) Other events in the regulation of Gad8 function: Gad8 shuttles between nucleus and cytoplasm, but the implications of its localization in its function and regulation have not been ascertained. Little is known about the dephosphorylation of the turn motif and the activation loop of Gad8 by protein phosphatases (depicted as PPase X and Y). (*5*) Other targets of PP2A-B55^Pab1^ are likely contributors to the transcriptional events brought about by the inactivation of PP2A-B55^Pab1^

Recent work in our laboratory identified the protein phosphatase PP2A-B55^Pab1^ as a key component in the regulation of TORC2 signaling during nutritional sensing (Martín et al. 2017). We observed that during nitrogen starvation the phosphorylation of the AKT homolog Gad8 (the substrate of TORC2) fluctuates, decreasing initially and then steadily increasing until it reaches a peak. This activation of the TORC2-Gad8 module is consistent with its essential role in the differentiation response. Interestingly, loss of PP2A-B55^Pab1^ mimics this increase in TORC2 signaling and is accompanied by an enhanced mating response. These findings point at PP2A-B55^Pab1^ being required to prevent untimely activity of the TORC2-Gad8 signaling module when in the presence of nitrogen. Yet, since the phosphorylation of Gad8 increases after nitrogen starvation, PP2A-B55^Pab1^ activity must itself be subject to regulation in this situation. The first indications of such regulation came from a study from the laboratory of Sergio Moreno. In budding yeast the kinase Rim15 and its substrates Igo1 and Igo2 (Greatwall and Endosulfins in higher eukaryotes) regulate entry into quiescence through PP2A-B55^Cdc55^ (Bontron et al. 2013). The Moreno laboratory showed that in fission yeast a similar regulation occurs during nitrogen starvation. In their study they showed that, in nitrogen rich conditions, TORC1 prevents the activity of the Rim15 homolog Ppk18 (Chica et al. 2016) (Fig. 1-1). Conversely, upon nitrogen depletion or TORC1 inactivation, Ppk18 gains activity, which results in the phosphorylation of Igo1. In its phosphorylated state, this protein acts as a potent inhibitor of PP2A-B55^Pab1^ (Williams et al. 2014). Hence, a decline in TORC1 activity promotes PP2A-B55^Pab1^ inactivation. This was shown to lead to a shortening of the G2 phase of the cell cycle and entry into mitosis. Thus, fission yeast cells connect nutritional sensing and growth to the cell cycle (Sveiczer and Horváth 2017) (Fig. 1-2).

We could confirm that inhibition of PP2A-B55^Pab1^ by Igo1 is also responsible for the increased phosphorylation of Gad8 Ser546 upon nitrogen starvation. In experiments where TORC1 activity was conditionally inactivated (*tor2*-*51*), phosphorylation of Gad8 Ser546 gradually increased in parallel to Igo1 phosphorylation. This was largely prevented in *igo1*-deleted cells (Martín et al. 2017). Therefore, PP2A-B55^Pab1^ acts as a mediator between the two TOR complexes. During growth on good nitrogen sources TORC1 through PP2A-B55^Pab1^ sends a message to TORC2, so that its activity is attenuated. When TORC1 activity decreases, indicating nitrogen scarcity in the medium, the reduction of PP2A-B55^Pab1^ activity conveys the opposite message to TORC2 resulting in the enhancement of Gad8 phosphorylation.

In addition to this regulation, studies from the Petersen laboratory have shown that the initial transient decrease in TORC2 activity during nitrogen stress enhances mitotic commitment through the regulation of the MAPK Sty1 (Petersen and Nurse 2007; Hartmuth and Petersen 2009). Therefore, a tight regulation of TORC2 signaling during nutritional sensing is key to achieve an adequate response.

## Future directions

In our work we have shown that PP2A-B55^Pab1^ can dephosphorylate Gad8 Ser546 (Fig. 1-3), and important event for the sexual differentiation response of fission yeast. Still, we cannot exclude other levels of regulation of Gad8, or the existence of additional substrates of PP2A-B55^Pab1^ that are also relevant to this process. We will discuss these and other interesting possibilities in this section.

### Additional levels of control of TORC2 and Gad8 activities

As a member of the AGC group of protein kinases, Gad8 is also phosphorylated in two other conserved phosphorylation sites (besides Ser546 in the hydrophobic motif): Ser527 in the turn motif and Thr387 in the activation loop (Matsuo et al. 2003). Whereas Ser527 is also phosphorylated by TORC2, Thr387 is phosphorylated by the PDK1 homolog Ksg1. Are these other sites also dynamically regulated during nitrogen starvation? We observed that mutation of Ser546 to Ala was alone sufficient to reduce Gad8 activity to a great extent, both in the presence or absence of PP2A-B55^Pab1^. Since phosphorylation of the hydrophobic motif of some AGC kinases creates a docking site for PDK1 (Matsuo et al. 2003; Pearce et al. 2010), altering the phosphorylation status of Ser546 most likely affects the phosphorylation of Thr387 and the overall activity of the protein. Whether PP2A-B55^Pab1^ can also dephosphorylate these other sites as part of a sequential reaction, or whether it might change the accessibility of other protein phosphatases, are hypotheses worth investigating as they might provide cues as to how Gad8 activity is fine-tuned in the cell.

To add a new layer of complexity, it has in recent years been shown that Gad8 can also regulate TORC2 through the phosphorylation of Tor1 Thr1972 in a negative feedback loop (Hálová et al. 2013). In addition, phosphorylation of Gad8 Thr6 impedes the interaction between Gad8 and TORC2, hence preventing the aforementioned feedback (Du et al. 2016). This intricate network of phosphorylation events present many new challenges to the field, including the identity of the counteracting phosphatases and their temporal and spatial regulation (Fig. 1-4).

TORC2 and Gad8 play important roles; not only during sexual differentiation but also in telomere maintenance and in the stress and DNA damage responses (Weisman and Choder 2001; Schonbrun et al. 2009). During an unperturbed cell cycle, those functions should not be inhibited by PP2A-B55^Pab1^, which poses the question of what limits its role to the differentiation functions of Gad8. Several explanations could account for this behavior: Different functions might require different thresholds of Gad8 activity, or it could be that only at certain locations is Gad8 subject to regulation by PP2A-B55^Pab1^. In the presence of PP2A-B55^Pab1^ activity (in nitrogen rich conditions) we could detect basal Gad8 Ser546 phosphorylation. This can be understood as the result of a lower catalytic efficiency in the dephosphorylation reaction compared to the phosphorylation by TORC2. Yet, it could also indicate that the entire population of Gad8 is not available for dephosphorylation by PP2A-B55^Pab1^. PP2A-B55^Pab1^ localizes throughout the cell, but it is enriched in the nucleus. Gad8 localization has been rather contentious in the field. Previous studies had shown it to be primarily cytoplasmic (Tatebe et al. 2010). However, recent work from the Weisman laboratory showed that Gad8 could also be found in the nucleus where it interacts with the MBF complex (Cohen et al. 2016). In our hands, Gad8 localizes predominantly to the cytoplasm. However, treatment with Leptomycin B leads to its accumulation in the nucleus, indicating that Gad8 shuttles between the two locations. Given the nuclear localization of PP2A-B55^Pab1^, an attractive hypothesis would be that only the pool of Gad8 in the nucleus becomes dephosphorylated by PP2A-B55^Pab1^. In the future, in vivo experiments where the localization of these proteins can be controlled will help to address this question (Fig. 1-4).

Finally, we cannot rule out that mechanisms of regulation other than phosphorylation control the activity of the TORC2-Gad8 module. For instance in budding yeast non-covalent binding of ubiquitin to Kog1 (the Raptor ortholog) protects it from degradation (Jiang 2016). Whether TORC2 can also be regulated in the same way has not been addressed yet.

### Extending the roles of PP2A-B55^Pab1^ and other protein phosphatases in differentiation

In our study we have identified Gad8 as an important substrate of PP2A-B55^Pab1^ in the regulation of sexual differentiation, but is it the only relevant one? Most likely there are other substrates of PP2A-B55^Pab1^ that also contribute to the phenotype of enhanced differentiation that we observed in a *pab1*Δ mutant. Mutation of Gad8 Ser546 to Ala completely blocked the mating response, both in the presence or absence of PP2A-B55^Pab1^. However, it reduced but did not completely block expression of *mei2*, suggesting that other proteins also contribute to this effect. In budding yeast, the Greatwall-Endosulfin pathway has been shown to regulate the expression of stress and stationary phase genes during a diauxic shift through the inhibition of PP2A-B55^Cdc55^ (Bontron et al. 2013). This is achieved via the regulation of the mRNA stability of the transcription factors Msn2/Msn4, and the activation of Gis1. Given the high degree of conservation of this pathway, it would not be surprising if in fission yeast PP2A-B55^Pab1^ could also regulate gene expression in a similar fashion. Future efforts will be dedicated to identifying new factors regulated by PP2A-B55^Pab1^ that contribute to the transcriptional program elicited during sexual differentiation (Fig. 1-5).

Finally, what other protein phosphatases participate in the differentiation response of fission yeast? Clearly, the Tap42 and Ppe1 roles are worth revisiting. Moreover, a second PP2A complex (PP2A-B56^Par1^) has also been identified in a screen for mutants resistant to TORC1 inhibition (Rallis et al. 2014). Altogether, these evidences point towards a complex and exciting scenario in the regulation and mediation of TOR signaling by protein phosphatases.

## Conclusions

The two TOR complexes are key mediators of nutritional signaling with seminal implications in cell differentiation, proliferation and metabolism. In this review, we have summarized their multiple functions and interplay, with special focus on the role of phosphatases in these events. Great challenges still lie ahead, but the latest findings bring us one step closer to fully comprehending the significance of protein phosphatases in the TOR-mediated cellular responses.
